# Phenyl *N*-phenyl­carbamate

**DOI:** 10.1107/S1600536809017887

**Published:** 2009-05-23

**Authors:** Durre Shahwar, M. Nawaz Tahir, M. Sharif Mughal, Muhammad Akmal Khan, Naeem Ahmad

**Affiliations:** a Department of Chemistry, Government College University, Lahore, Pakistan; b Department of Physics, University of Sargodha, Pakistan; c Department of Zoology, Government College University, Lahore, Pakistan

## Abstract

In the title compound, C_13_H_11_NO_2_, the aromatic rings are oriented at a dihedral angle of 42.52 (12)°. The crystal structure is stabilized by inter­molecular N—H⋯O hydrogen bonds, which form infinite one-dimensional polymeric chains extending along the *a* axis. C—H⋯π inter­actions between the aromatic rings are also present.

## Related literature

For related structures, see: Haufe *et al.* (2003 ▶); Shah *et al.* (2008 ▶, 2009 ▶); Xu & Qu (2008 ▶).
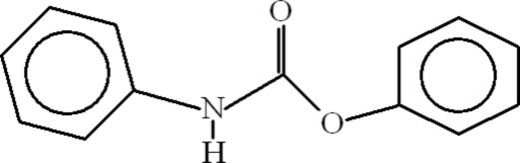


## Experimental

### 

#### Crystal data

C_13_H_11_NO_2_
*M* *_r_* = 213.23
Orthorhombic, 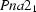

*a* = 9.4734 (9) Å
*b* = 19.5825 (17) Å
*c* = 5.8509 (5) Å
*V* = 1085.42 (17) Å^3^
*Z* = 4
Mo *K*α radiation
μ = 0.09 mm^−1^
*T* = 296 K
0.22 × 0.12 × 0.12 mm

#### Data collection

Bruker Kappa APEXII CCD diffractometer
Absorption correction: multi-scan (*SADABS*; Bruker, 2005 ▶) *T*_min_ = 0.985, *T*_max_ = 0.988
6579 measured reflections
1505 independent reflections
751 reflections with *I* > 2σ(*I*)
*R*_int_ = 0.071

#### Refinement

*R*[*F* ^2^ > 2σ(*F* ^2^)] = 0.046
*wR*(*F* ^2^) = 0.086
*S* = 0.97
1505 reflections
145 parameters
1 restraint
H-atom parameters constrained
Δρ_max_ = 0.13 e Å^−3^
Δρ_min_ = −0.17 e Å^−3^

### 

Data collection: *APEX2* (Bruker, 2007 ▶); cell refinement: *SAINT* (Bruker, 2007 ▶); data reduction: *SAINT*; program(s) used to solve structure: *SHELXS97* (Sheldrick, 2008 ▶); program(s) used to refine structure: *SHELXL97* (Sheldrick, 2008 ▶); molecular graphics: *ORTEP-3 for Windows* (Farrugia, 1997 ▶) and *PLATON* (Spek, 2009 ▶); software used to prepare material for publication: *WinGX* (Farrugia, 1999 ▶) and *PLATON*.

## Supplementary Material

Crystal structure: contains datablocks global, I. DOI: 10.1107/S1600536809017887/at2785sup1.cif

Structure factors: contains datablocks I. DOI: 10.1107/S1600536809017887/at2785Isup2.hkl

Additional supplementary materials:  crystallographic information; 3D view; checkCIF report

## Figures and Tables

**Table 1:** Hydrogen-bond geometry (Å, °)

*D*—H⋯*A*	*D*—H	H⋯*A*	*D*⋯*A*	*D*—H⋯*A*
N1—H1⋯O2^i^	0.86	2.14	2.976 (3)	165
C3—H3⋯*Cg*2^ii^	0.93	2.80	3.673 (4)	156
C10—H10⋯*Cg*1^iii^	0.93	2.86	3.599 (4)	137

Symmetry codes: (i) 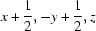; (ii) 
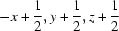
; (iii) 
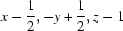
. *Cg*1 and *Cg*2 are the centroids of the C1–C6 and C8–C13 rings, respectively.

**Fig. 1.**
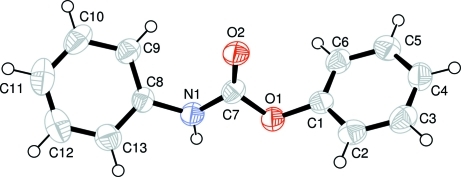
View of the title compound with the atom numbering scheme. The thermal ellipsoids are drawn at the 50% probability level. H-atoms are shown as small circles of arbitrary radii.

**Fig. 2.**
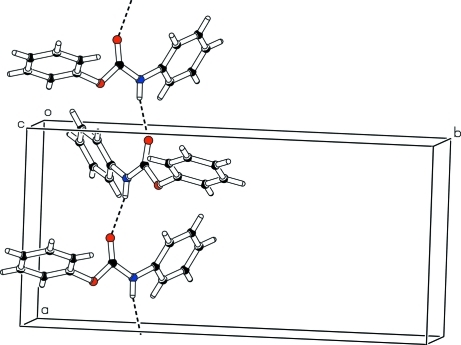
A partial packing diagram showing infinite one-dimensional chains along the a axis.

## supplementary crystallographic information

### Comment

The title compound (I), (Fig. 1), is synthesized for investigation of biological activity like enzyme inhibition and antimicrobial activity. It is one of the members of a series of carbamates being synthesized in our laboratory.

Various crystal structures of *N*-phenylcarbamates with different attachments have been reported. Examples include (II) 4-nitrophenyl *N*-phenylcarbamate (Xu & Qu, 2008), (III) *cis*-4-fluorocyclohexyl *N*-phenylcarbamate, *cis*-4-hydroxycyclohexyl *N*-phenylcarbamate and 4-oxocyclohexyl *N*-phenylcarbamate (Haufe *et al.*, 2003). The title compound is a *N*-phenylcarbamate with the simplest type of aromatic ring. In (I), the rings A (C1—C6) and B (C8—C13) are oriented at a dihedral angle of 42.49 (13)°. The title compound is stabilized in the form of infinite one-dimensional polymeric chains due to intermolecular N—H···O H-bonding. These chains extend along the crystallographic *a* axis (Table 1, Fig. 2). Similar infinite chains also due to intermolecular N—H···O H-bonding have also been found in 3-[(3,4-dichlorophenyl)aminocarbonyl]-propionic acid (Shah *et al.*, 2009), 4-[(2-fluorophenyl)amino]-4-oxobutanoic acid (Shah *et al.*, 2008). The packing may also be stabilized due to C—H···π interactions (Table 1).

### Experimental

A solution of aniline (0.913 ml, 0.01 mol) and dichloromethane (20 ml) was prepared. Phenylchloroformate (1.26 ml, 0.01 mol) was added dropwise to the magnetically stirring solution. The mixture turned to a suspension after 1 h due to stirring at room temperature. To obtain the final product, n-hexane (30 ml) was added and a precipitate was formed. The precipitate was filtered and  recrystalized from ethylacetate and methanol (9:1).

### Refinement

In the absence of significant anomalous scattering effects, Friedel pairs were merged. H-atoms were positioned geometrically, with N—H = 0.86 Å and C—H = 0.93 Å for aromatic rings and constrained to ride on their parent atoms, with U_iso_(H) = xU_eq_(C, N), where x = 1.2 for all H atoms.

### Crystal data

**Table:**

C_13_H_11_NO_2_	*F*(000) = 448
*M**_r_* = 213.23	*D*_x_ = 1.305 Mg m^−^^3^
Orthorhombic, *P**n**a*2_1_	Mo *K*α radiation, λ = 0.71073 Å
Hall symbol: P 2c -2n	Cell parameters from 2241 reflections
*a* = 9.4734 (9) Å	θ = 3.0–28.6°
*b* = 19.5825 (17) Å	µ = 0.09 mm^−^^1^
*c* = 5.8509 (5) Å	*T* = 296 K
*V* = 1085.42 (17) Å^3^	Needle, colourless
*Z* = 4	0.22 × 0.12 × 0.12 mm

### Data collection

**Table:**

Bruker Kappa APEXII CCD diffractometer	1505 independent reflections
Radiation source: fine-focus sealed tube	751 reflections with *I* > 2σ(*I*)
graphite	*R*_int_ = 0.071
Detector resolution: 7.40 pixels mm^-1^	θ_max_ = 28.6°, θ_min_ = 3.0°
ω scans	*h* = −12→12
Absorption correction: multi-scan (*SADABS*; Bruker, 2005)	*k* = −26→26
*T*_min_ = 0.985, *T*_max_ = 0.988	*l* = −4→7
6579 measured reflections	

### Refinement

**Table:**

Refinement on *F*^2^	Primary atom site location: structure-invariant direct methods
Least-squares matrix: full	Secondary atom site location: difference Fourier map
*R*[*F*^2^ > 2σ(*F*^2^)] = 0.046	Hydrogen site location: inferred from neighbouring sites
*wR*(*F*^2^) = 0.086	H-atom parameters constrained
*S* = 0.97	*w* = 1/[σ^2^(*F*_o_^2^) + (0.028*P*)^2^] where *P* = (*F*_o_^2^ + 2*F*_c_^2^)/3
1505 reflections	(Δ/σ)_max_ < 0.001
145 parameters	Δρ_max_ = 0.13 e Å^−^^3^
1 restraint	Δρ_min_ = −0.16 e Å^−^^3^

### Special details

**Table:**

Geometry. Bond distances, angles *etc*. have been calculated using the rounded fractional coordinates. All su's are estimated from the variances of the (full) variance-covariance matrix. The cell e.s.d.'s are taken into account in the estimation of distances, angles and torsion angles
Refinement. Refinement of *F*^2^ against ALL reflections. The weighted *R*-factor *wR* and goodness of fit *S* are based on *F*^2^, conventional *R*-factors *R* are based on *F*, with *F* set to zero for negative *F*^2^. The threshold expression of *F*^2^ > σ(*F*^2^) is used only for calculating *R*-factors(gt) *etc*. and is not relevant to the choice of reflections for refinement. *R*-factors based on *F*^2^ are statistically about twice as large as those based on *F*, and *R*- factors based on ALL data will be even larger.

### Fractional atomic coordinates and isotropic or equivalent isotropic displacement parameters (Å^2^)

**Table:**

	*x*	*y*	*z*	*U*_iso_*/*U*_eq_	
O1	0.2597 (2)	0.32604 (11)	0.9484 (4)	0.0567 (9)	
O2	0.0419 (2)	0.29274 (9)	0.8340 (5)	0.0516 (8)	
N1	0.2427 (3)	0.23828 (12)	0.7187 (5)	0.0452 (10)	
C1	0.2055 (3)	0.37847 (16)	1.0868 (7)	0.0443 (14)	
C2	0.2411 (4)	0.44407 (15)	1.0327 (7)	0.0510 (14)	
C3	0.2016 (4)	0.49574 (16)	1.1764 (7)	0.0573 (15)	
C4	0.1281 (4)	0.48187 (15)	1.3717 (7)	0.0553 (14)	
C5	0.0927 (4)	0.41549 (16)	1.4235 (7)	0.0550 (14)	
C6	0.1333 (4)	0.36338 (15)	1.2818 (7)	0.0489 (13)	
C7	0.1675 (3)	0.28518 (15)	0.8343 (7)	0.0422 (11)	
C8	0.1863 (3)	0.19143 (14)	0.5605 (7)	0.0414 (13)	
C9	0.0627 (3)	0.15661 (14)	0.5992 (6)	0.0486 (13)	
C10	0.0146 (4)	0.11085 (16)	0.4366 (8)	0.0633 (18)	
C11	0.0904 (5)	0.09913 (18)	0.2388 (8)	0.0700 (16)	
C12	0.2135 (5)	0.13340 (19)	0.2040 (7)	0.0667 (18)	
C13	0.2617 (4)	0.17989 (15)	0.3606 (7)	0.0548 (13)	
H1	0.33213	0.23677	0.74330	0.0544*	
H2	0.29156	0.45348	0.90007	0.0616*	
H3	0.22491	0.54063	1.14082	0.0689*	
H4	0.10216	0.51715	1.46947	0.0664*	
H5	0.04108	0.40599	1.55489	0.0657*	
H6	0.11184	0.31833	1.31818	0.0584*	
H9	0.01197	0.16370	0.73313	0.0581*	
H10	−0.06985	0.08773	0.46083	0.0757*	
H11	0.05769	0.06814	0.13063	0.0838*	
H12	0.26557	0.12516	0.07208	0.0799*	
H13	0.34490	0.20372	0.33304	0.0658*	

### Atomic displacement parameters (Å^2^)

**Table:**

	*U*^11^	*U*^22^	*U*^33^	*U*^12^	*U*^13^	*U*^23^
O1	0.0340 (14)	0.0611 (13)	0.0751 (19)	−0.0041 (12)	0.0042 (13)	−0.0266 (14)
O2	0.0283 (12)	0.0562 (12)	0.0702 (18)	0.0033 (11)	−0.0034 (13)	−0.0119 (12)
N1	0.0287 (15)	0.0489 (15)	0.058 (2)	0.0041 (14)	−0.0041 (15)	−0.0099 (15)
C1	0.032 (2)	0.052 (2)	0.049 (3)	−0.0012 (16)	−0.003 (2)	−0.0058 (19)
C2	0.050 (2)	0.054 (2)	0.049 (3)	−0.0063 (18)	0.000 (2)	0.0073 (19)
C3	0.059 (3)	0.0410 (19)	0.072 (3)	−0.0043 (17)	−0.002 (2)	0.006 (2)
C4	0.053 (2)	0.049 (2)	0.064 (3)	0.0016 (17)	−0.001 (2)	−0.0118 (19)
C5	0.055 (2)	0.059 (2)	0.051 (3)	−0.0031 (17)	0.006 (2)	0.001 (2)
C6	0.048 (2)	0.0386 (17)	0.060 (3)	−0.0006 (16)	−0.002 (2)	0.0033 (19)
C7	0.040 (2)	0.0385 (15)	0.048 (2)	−0.0018 (17)	−0.007 (2)	0.0020 (17)
C8	0.039 (2)	0.0362 (17)	0.049 (3)	0.0063 (15)	−0.006 (2)	−0.0044 (17)
C9	0.043 (2)	0.0437 (17)	0.059 (3)	−0.0001 (16)	−0.0045 (19)	−0.0024 (18)
C10	0.049 (3)	0.053 (2)	0.088 (4)	−0.0040 (18)	−0.021 (3)	−0.008 (2)
C11	0.079 (3)	0.062 (2)	0.069 (3)	0.014 (2)	−0.024 (3)	−0.022 (2)
C12	0.077 (4)	0.069 (2)	0.054 (3)	0.019 (2)	−0.002 (3)	−0.012 (2)
C13	0.052 (2)	0.0485 (18)	0.064 (3)	0.0044 (17)	0.007 (2)	−0.004 (2)

### Geometric parameters (Å, °)

**Table:**

O1—C1	1.405 (4)	C9—C10	1.384 (5)
O1—C7	1.360 (4)	C10—C11	1.381 (6)
O2—C7	1.199 (3)	C11—C12	1.361 (6)
N1—C7	1.345 (4)	C12—C13	1.370 (5)
N1—C8	1.409 (4)	C2—H2	0.9300
N1—H1	0.8600	C3—H3	0.9300
C1—C2	1.365 (4)	C4—H4	0.9300
C1—C6	1.363 (5)	C5—H5	0.9300
C2—C3	1.368 (5)	C6—H6	0.9300
C3—C4	1.365 (6)	C9—H9	0.9300
C4—C5	1.376 (4)	C10—H10	0.9300
C5—C6	1.370 (5)	C11—H11	0.9300
C8—C13	1.389 (5)	C12—H12	0.9300
C8—C9	1.374 (4)	C13—H13	0.9300
			
O2···C6	3.087 (5)	C10···H3^ix^	3.0700
O2···C9	3.005 (4)	C13···H6^iii^	3.0700
O2···N1^i^	2.976 (3)	H1···H13	2.4900
O1···H9^ii^	2.7100	H1···O2^ii^	2.1400
O2···H5^iii^	2.7500	H1···H9^ii^	2.5900
O2···H9	2.6100	H3···C9^x^	3.0400
O2···H1^i^	2.1400	H3···C10^x^	3.0700
N1···O2^ii^	2.976 (3)	H4···H12^xi^	2.5300
C1···C10^iv^	3.579 (5)	H5···O2^v^	2.7500
C5···C7^v^	3.576 (5)	H5···C3^xii^	3.0800
C6···O2	3.087 (5)	H6···C7	2.9500
C6···C7^v^	3.592 (6)	H6···C8^v^	2.9500
C7···C6^iii^	3.592 (6)	H6···C13^v^	3.0700
C7···C5^iii^	3.576 (5)	H6···H13^viii^	2.5700
C9···O2	3.005 (4)	H9···O2	2.6100
C10···C1^vi^	3.579 (5)	H9···C7	2.8600
C2···H11^iv^	3.0600	H9···O1^i^	2.7100
C3···H5^vii^	3.0800	H9···H1^i^	2.5900
C6···H13^viii^	3.0500	H11···C2^vi^	3.0600
C7···H9	2.8600	H12···H4^xiii^	2.5300
C7···H6	2.9500	H13···H1	2.4900
C8···H6^iii^	2.9500	H13···C6^xiv^	3.0500
C9···H3^ix^	3.0400	H13···H6^xiv^	2.5700
			
C1—O1—C7	118.6 (2)	C8—C13—C12	120.0 (3)
C7—N1—C8	125.1 (3)	C1—C2—H2	120.00
C7—N1—H1	117.00	C3—C2—H2	120.00
C8—N1—H1	117.00	C2—C3—H3	120.00
O1—C1—C2	117.6 (3)	C4—C3—H3	120.00
O1—C1—C6	120.5 (3)	C3—C4—H4	120.00
C2—C1—C6	121.5 (3)	C5—C4—H4	120.00
C1—C2—C3	119.1 (4)	C4—C5—H5	120.00
C2—C3—C4	120.5 (3)	C6—C5—H5	120.00
C3—C4—C5	119.8 (3)	C1—C6—H6	120.00
C4—C5—C6	120.1 (4)	C5—C6—H6	120.00
C1—C6—C5	119.1 (3)	C8—C9—H9	120.00
O1—C7—O2	124.4 (3)	C10—C9—H9	120.00
O1—C7—N1	108.0 (2)	C9—C10—H10	120.00
O2—C7—N1	127.5 (3)	C11—C10—H10	120.00
N1—C8—C9	122.6 (3)	C10—C11—H11	120.00
C9—C8—C13	119.8 (3)	C12—C11—H11	120.00
N1—C8—C13	117.7 (3)	C11—C12—H12	120.00
C8—C9—C10	119.3 (3)	C13—C12—H12	120.00
C9—C10—C11	120.8 (3)	C8—C13—H13	120.00
C10—C11—C12	119.3 (4)	C12—C13—H13	120.00
C11—C12—C13	120.9 (4)		
			
C7—O1—C1—C2	−119.0 (3)	C1—C2—C3—C4	0.3 (6)
C7—O1—C1—C6	68.1 (4)	C2—C3—C4—C5	−0.4 (6)
C1—O1—C7—O2	3.9 (5)	C3—C4—C5—C6	1.2 (6)
C1—O1—C7—N1	−178.6 (3)	C4—C5—C6—C1	−1.8 (6)
C8—N1—C7—O1	−172.7 (3)	N1—C8—C9—C10	−179.4 (3)
C7—N1—C8—C13	138.2 (3)	C13—C8—C9—C10	−0.6 (5)
C8—N1—C7—O2	4.7 (6)	N1—C8—C13—C12	178.2 (3)
C7—N1—C8—C9	−43.0 (5)	C9—C8—C13—C12	−0.7 (5)
O1—C1—C2—C3	−173.7 (3)	C8—C9—C10—C11	1.1 (5)
C2—C1—C6—C5	1.6 (6)	C9—C10—C11—C12	−0.3 (6)
C6—C1—C2—C3	−0.9 (6)	C10—C11—C12—C13	−1.0 (6)
O1—C1—C6—C5	174.2 (3)	C11—C12—C13—C8	1.5 (6)

Symmetry codes: (i) *x*−1/2, −*y*+1/2, *z*; (ii) *x*+1/2, −*y*+1/2, *z*; (iii) *x*, *y*, *z*−1; (iv) *x*+1/2, −*y*+1/2, *z*+1; (v) *x*, *y*, *z*+1; (vi) *x*−1/2, −*y*+1/2, *z*−1; (vii) −*x*, −*y*+1, *z*−1/2; (viii) *x*−1/2, −*y*+1/2, *z*+1; (ix) −*x*+1/2, *y*−1/2, *z*−1/2; (x) −*x*+1/2, *y*+1/2, *z*+1/2; (xi) −*x*+1/2, *y*+1/2, *z*+3/2; (xii) −*x*, −*y*+1, *z*+1/2; (xiii) −*x*+1/2, *y*−1/2, *z*−3/2; (xiv) *x*+1/2, −*y*+1/2, *z*−1.

### Hydrogen-bond geometry (Å, °)

**Table:**

*D*—H···*A*	*D*—H	H···*A*	*D*···*A*	*D*—H···*A*
N1—H1···O2^ii^	0.8600	2.1400	2.976 (3)	165.00
C3—H3···Cg2^x^	0.9300	2.8000	3.673 (4)	156.00
C10—H10···Cg1^vi^	0.9300	2.8600	3.599 (4)	137.00

Symmetry codes: (ii) *x*+1/2, −*y*+1/2, *z*; (x) −*x*+1/2, *y*+1/2, *z*+1/2; (vi) *x*−1/2, −*y*+1/2, *z*−1.
